# Fungal Degradation of Extractives Plays an Important Role in the Brown Rot Decay of Scots Pine Heartwood

**DOI:** 10.3389/fpls.2022.912555

**Published:** 2022-05-12

**Authors:** Tiina Belt, Anni Harju, Petri Kilpeläinen, Martti Venäläinen

**Affiliations:** ^1^Production Systems Unit, Biomass Characterization and Properties, Natural Resources Institute Finland, Espoo, Finland; ^2^Production Systems Unit, Biomass Characterization and Properties, Natural Resources Institute Finland, Savonlinna, Finland; ^3^Production Systems Unit, Biorefinery and Bioproducts, Natural Resources Institute Finland, Espoo, Finland

**Keywords:** brown rot, *Coniophora puteana*, heartwood, hydroxylation, resin acid, *Rhodonia placenta*, stilbene, pinosylvin

## Abstract

Scots pine heartwood is known to have resistance to wood decay due to the presence of extractives, namely stilbenes and resin acids. However, previous studies have indicated that these extractives are degradable by wood decaying fungi. This study aimed to investigate the relationship between extractive degradation and heartwood decay in detail and to gain insight into the mechanisms of extractive degradation. Mass losses recorded after a stacked-sample decay test with brown rot fungi showed that the heartwood had substantial decay resistance against *Coniophora puteana* but little resistance against *Rhodonia placenta*. Extracts obtained from the decayed heartwood samples revealed extensive degradation of stilbenes by *R. placenta* in the early stages of decay and a noticeable but statistically insignificant loss of resin acids. The extracts from *R. placenta*-degraded samples contained new compounds derived from the degraded extractives: hydroxylated stilbene derivatives appeared in the early decay stages and then disappeared, while compounds tentatively identified as hydroxylated derivatives of dehydroabietic acid accumulated in the later stages. The degradation of extractives was further analysed using simple degradation assays where an extract obtained from intact heartwood was incubated with fungal mycelium or extracellular culture fluid from liquid fungal cultures or with neat Fenton reagent. The assays showed that extractives can be eliminated by several fungal degradative systems and revealed differences between the degradative abilities of the two fungi. The results of the study indicate that extractive degradation plays an important role in heartwood decay and highlight the complexity of the fungal degradative systems.

## Introduction

Wood decaying fungi are natural decomposers that have specialised in degrading and consuming the polymeric constituents of wood. In nature, wood decaying fungi serve an important ecological role in carbon and nutrient recycling (Watkinson et al., 2006), but they can cause significant damage in the built environment by degrading wood in service (Schmidt, 2007). Given the economic losses caused by wood decay and the safety hazards posed by degrading wood structures, wood decay, and its mechanisms, prediction and prevention remain topics of active interest (Ringman et al., 2019; Goodell et al., 2020; Brischke et al., 2021).

Wood decaying fungi are traditionally classified as brown rot, white rot, or soft rot depending on the type of decay they cause. Brown rots degrade wood by a two-step mechanism: the structure of the wood material is first disrupted using hydroxyl radicals derived from a biological chelator-mediated Fenton reaction, after which the cell wall carbohydrates are hydrolysed to digestible sugars using carbohydrate active enzymes (Arantes et al., 2012; Zhang et al., 2016). The Fenton reaction takes place within the wood cell wall, causing the depolymerisation and rapid repolymerisation of lignin (Yelle et al., 2008, 2011) and the depolymerisation of the cell wall carbohydrates (Kleman-Leyer et al., 1992; Goodell et al., 2017). In addition to degradative agents targeting the wood cell wall polymers, brown rots and other wood decaying fungi have also evolved comprehensive detoxification systems to deal with toxic organic compounds present in the substrate or arising from its breakdown. These systems include the intracellular detoxification system composed of cytochrome P450 monooxygenases and transferases, and various extracellular enzymes that can assist in detoxification such as laccases and peroxidases (Morel et al., 2013; Kües, 2015).

Despite the highly evolved and purpose-built degradative systems of brown rots and other wood decaying fungi, the heartwoods produced by some tree species have natural resistance towards degradation. The durability of heartwoods is generally attributed to the presence of antifungal heartwood extractives, but in above-ground applications with intermittent drying and wetting their resistance is also influenced by water exclusion efficiency (Meyer-Veltrup et al., 2017). The durability of Scots pine is typically rated as “moderate” or “slight” (Van Acker et al., 2003; EN 350, 2016), and it is primarily attributed to two classes of extractives, the stilbenes and resin acids (Harju et al., 2002, 2003; Venäläinen et al., 2003, 2004). The resistance of heartwood is particularly strongly correlated with its stilbene content, but post-decay extractive content measurements have shown that stilbenes are actually degradable by brown rot fungi in both natural heartwood (Karppanen et al., 2008; Belt et al., 2019) and wood impregnated with heartwood stilbenes (Lu et al., 2016). Resin acids may also be degraded (Karppanen et al., 2008; Belt et al., 2019). Although the degradation of heartwood extractives has been demonstrated, the relationship between wood degradation and extractive degradation has not been thoroughly explored. Furthermore, although some stilbene degradation products were identified in stilbene-impregnated wood (Lu et al., 2016), the degradation products of natural heartwood remain uncharacterised, and the fungal degradative systems involved in degradation remain unknown.

The purpose of the present study was to investigate the fungal degradation of Scots pine (*Pinus sylvestris* L.) heartwood extractives in detail. Small blocks of pine heartwood were exposed to the brown rot fungi *Coniophora puteana* (Schum. ex Fries) Karst. and *Rhodonia placenta* (Fr.) Niemelä, K. H. Larsson & Schigel in a stacked-sample decay test designed to produce a series of samples in different stages of decay. To gain insight into the mechanisms of wood decay and extractive degradation, the mass losses and extractive contents of the blocks were measured along the sample stack, and the emerging degradation products were identified (when possible). Fluorescence images were also collected from the blocks to visualise the degradation of extractives at the cellular level. Finally, the degradation of extractives was studied using simple assays to explore the involvement of fungal intracellular and extracellular enzymes and the Fenton reaction. The results provide insight into the role of extractive degradation in heartwood decay and highlight the complexity of the fungal degradative systems and the differences in degradative capability between fungi.

## Materials and Methods

### Wood Material Preparation

All wood materials were prepared from one log cut from an approximately 70-year-old Scots pine tree grown in Ruotsinkylä, Finland (N 60°36′, E 26°27′). The log was stored frozen and protected from light and desiccation until use. For decay tests, pine sapwood and heartwood sample blocks, with dimensions 12 mm (radial) × 8 mm (tangential) × 8 mm (longitudinal), were prepared from one thick disc. The blocks were prepared by first sawing long longitudinal slats 12 mm in width (radial) and 8 mm in depth (tangential) from the disc and then cutting the slats in length to produce the final 8 mm long samples. To minimise radial variations in heartwood extractive composition, all heartwood samples were cut from the same radial position (=starting from the same annual ring). Outer heartwood was used due to its higher expected stilbene content (Venäläinen et al., 2003; Belt et al., 2020). All sapwood samples were cut from middle sapwood (annual ring not specified). The heartwood and sapwood samples were dried at 60°C for 24 h, weighed to determine their initial weight and then sterilised by ionizing radiation (25–50 kGy dose). Samples dried at 60°C are expected to contain 1%–2% of residual moisture. Gamma sterilisation was used instead of steam to avoid the potential negative effects of moisture and high temperatures on extractives.

For extract degradation assays, additional wood materials were obtained from sapwood and outer heartwood (annual ring positions not specified), ground to a fine powder in a laboratory mill and dried at 60°C. A portion of the heartwood powder was extracted in an accelerated solvent extractor (Dionex ASE 350) in 3 × 5 min cycles at 90°C using methanol as solvent. The solvent was removed, and the extract redissolved in acetone at 2.5 mg/ml.

### Decay Test

**Figure 1:**
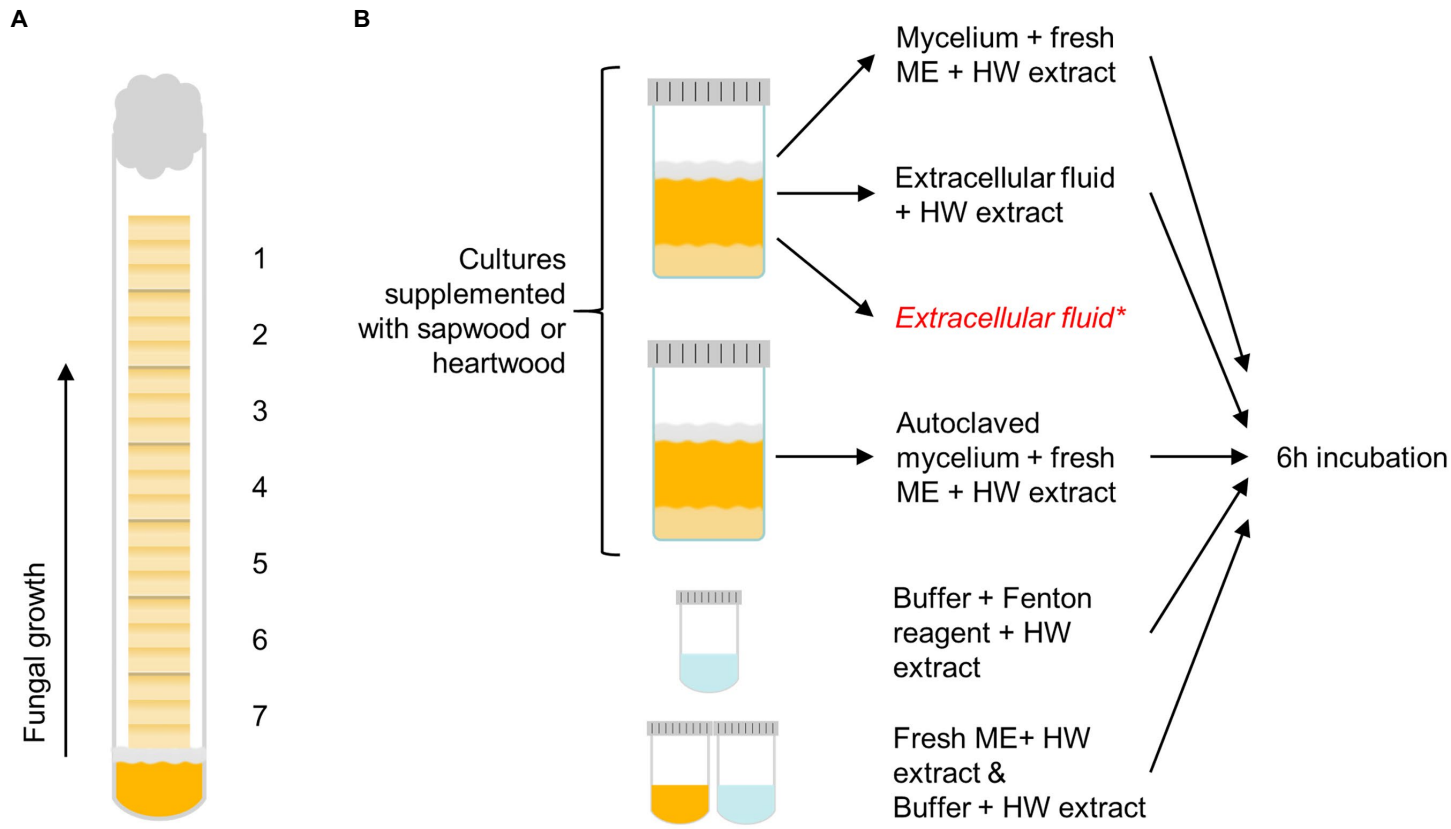
The decay test set-up with sample positions 1–7 labelled **(A)** and the outline of the extract degradation assays **(B)**. Pine heartwood (HW) extract was incubated with mycelium or extracellular fluid from *Coniophora puteana* and *Rhodonia placenta* liquid cultures (supplemented with sapwood or heartwood powder), or with Fenton reagent. Extract was also incubated with autoclaved mycelium, fresh malt extract broth (ME), or buffer to act as references for the mycelium, extracellular fluid, and Fenton reagent incubations, respectively. *An additional control sample of extracellular fluid was collected from heartwood cultures and analysed immediately without incubation to determine the concentration of solubilised extractives.

The sapwood and heartwood sample blocks were exposed to the brown rot fungi *C. puteana* (strain BAM Ebw. 15) and *R. placenta* (strain BAM 113) in test tubes (16 mm diameter) containing 4 ml of 2% malt extract agar. Each test tube was inoculated with a plug of mycelium from the growing edge of a fungal stock culture maintained on 2% malt extract agar. Seven sterile wood samples were stacked in the test tubes over a sterilised piece of plastic netting, which was placed between the mycelial plug and the samples to prevent the absorption of water from the agar. Each tube received seven consecutive samples from one slat. Six replicate tubes of heartwood and sapwood were prepared per fungus. The prepared tubes were plugged with cotton wads and cultivated at 85% RH and 20°C in the dark. The test was run for 5 months until the visible mycelial front of one fungus reached the top of the topmost sample in one tube. The decay test set-up is depicted in Figure 1A.

After the decay test, one of the six replicate tubes was taken aside for imaging, while the samples in the remaining five replicate tubes were removed from the tubes, gently brushed to remove adhered mycelium, airdried in a fume hood for 24 h, and then finally dried at 60°C for 24 h to determine their decayed weight. Mass loss was calculated by dividing the difference between initial and decayed dry mass with the initial dry mass.

### Fluorescence Imaging

Using a rotary microtome, 25-μm-thick transverse and radial sections were cut from the samples set aside for imaging. The sections were embedded in water on objective slides, covered with coverslips, and edge-sealed with nail polish. The fluorescence imaging systems consisted of an Olympus BX53 microscope, a UV excitation filter set (330–385 nm excitation filter, 420 nm long pass emission filter), a 4x (NA 0.13) or 20x (NA 0.5) air objective, and a QImaging Micropublisher RTV 3.3 camera. UV excitation was used because it is known to induce fluorescence in both stilbenes and resin acids (Donaldson et al., 2019; Belt et al., 2020, 2021). The 20x magnification images were collected after 30 s of exposure to UV light in the microscope to induce changes in fluorescence that allowed the visualisation of changes due to heartwood decay.

### Heartwood Extractive Analysis

After weighing, the decayed heartwood samples were individually ground to a fine powder in a laboratory mill. Portions (10 mg) of each wood powder were extracted with 2 ml of MeOH (30 μg/ml of heptadecanoic acid as internal standard) for 30 min in a sonicator. An aliquot (750 μl) of each extract was evaporated to dryness under nitrogen, redissolved in 100 μl of N,O-bis(trimethylsilyl)trifluoroacetamide, 50 μl of chlorotrimethylsilane and 20 μl of pyridine, and then trimethylsilylated at 70°C for 20 min. The samples were analysed by GC–MS on a HP 6890/HP 5973 system, using a HP-5 column (30 m; 0.25 inner diameter, 0.25 μm film thickness), helium as carrier gas (1.5 ml/min) and the following oven temperature program: 18°C/min from 170 to 220°C (3 min), 10°C/min from 220 to 260°C, and 5°C/min from 260 to 300°C.

### Extractive Degradation Assays

The degradation of extractives was studied by incubating the heartwood extract (section Wood Material Preparation) with fungal mycelium, extracellular culture fluid, or neat Fenton reagent (see Figure 1B). To produce the mycelium and culture fluid, *C. puteana* and *R. placenta* were grown in 10 ml liquid cultures containing 2% (w/v) of malt extract and 5% (w/v) of sapwood or heartwood powder. The cultures were inoculated with one plug of mycelium from the growing edge of a fungal stock culture maintained on 2% malt extract agar and then incubated at room temperature for 13–25 days. Ten replicates were prepared per culture type (40 cultures in total). Four milliliter of culture fluid was collected from five replicate cultures, while the mycelial mat from the same cultures was moved to new a container with 10 ml of fresh malt extract broth. An additional 3-ml control sample of extracellular fluid was collected from the heartwood cultures to determine the concentration of solubilised extractives in the fluid. Mycelial mats were also collected from the remaining five cultures, moved to fresh malt extract broth, and then autoclaved. The simple Fenton reaction was prepared in 50 mM acetate buffer (pH 4.0) and contained 10 mM of FeCl_2_ and 100 mM of H_2_O_2_.

To assay the degradation of extractives, heartwood extract (section Wood Material Preparation) was added to the fungal mycelia in malt extract broth, to the 4-ml samples of extracellular fluid, and to the simple Fenton reagent solutions to a final concentration of 50 μg/ml. Extract was also added to the autoclaved mycelia in malt extract broth and to plain malt extract and plain acetate buffer to act as references for the mycelium, extracellular fluid, and Fenton reaction incubations, respectively. No extract was added to the additional 3-ml control samples of extracellular fluid from the heartwood cultures. All the prepared reactions with added heartwood extract were incubated at room temperature for 6 h protected from light, after which 4 ml of each solution was collected and extracted with 3 × 2 ml of ethyl acetate to separate the extractives from the aqueous solution. The additional 3-ml control samples of extracellular fluid were extracted immediately without incubation. The combined ethyl acetate phases from each extraction were evaporated to dryness under nitrogen and then trimethylsilylated and analysed by GC–MS as above (section Heartwood Extractive Analysis) to determine the changes in extractive concentration. The concentration of extractives in the 3-ml control sample of heartwood culture fluid was subtracted from the final concentration in the heartwood culture fluid incubation.

### Statistical Analysis

The normality of the data was checked using the Shapiro–Wilk test. The statistical significance of the changes in stilbene and resin acid content in decayed heartwood was tested using Kruskal-Wallis one-way ANOVA, while the statistical significance of the changes in stilbene and resin acid concentration in the extractive degradation assays was tested using Student’s *t*-test. All statistical calculations were performed in SigmaPlot 14.

## Results

### Stacked-Sample Decay Test

#### Mass Losses of Scots Pine Sapwood and Heartwood

**Figure 2:**
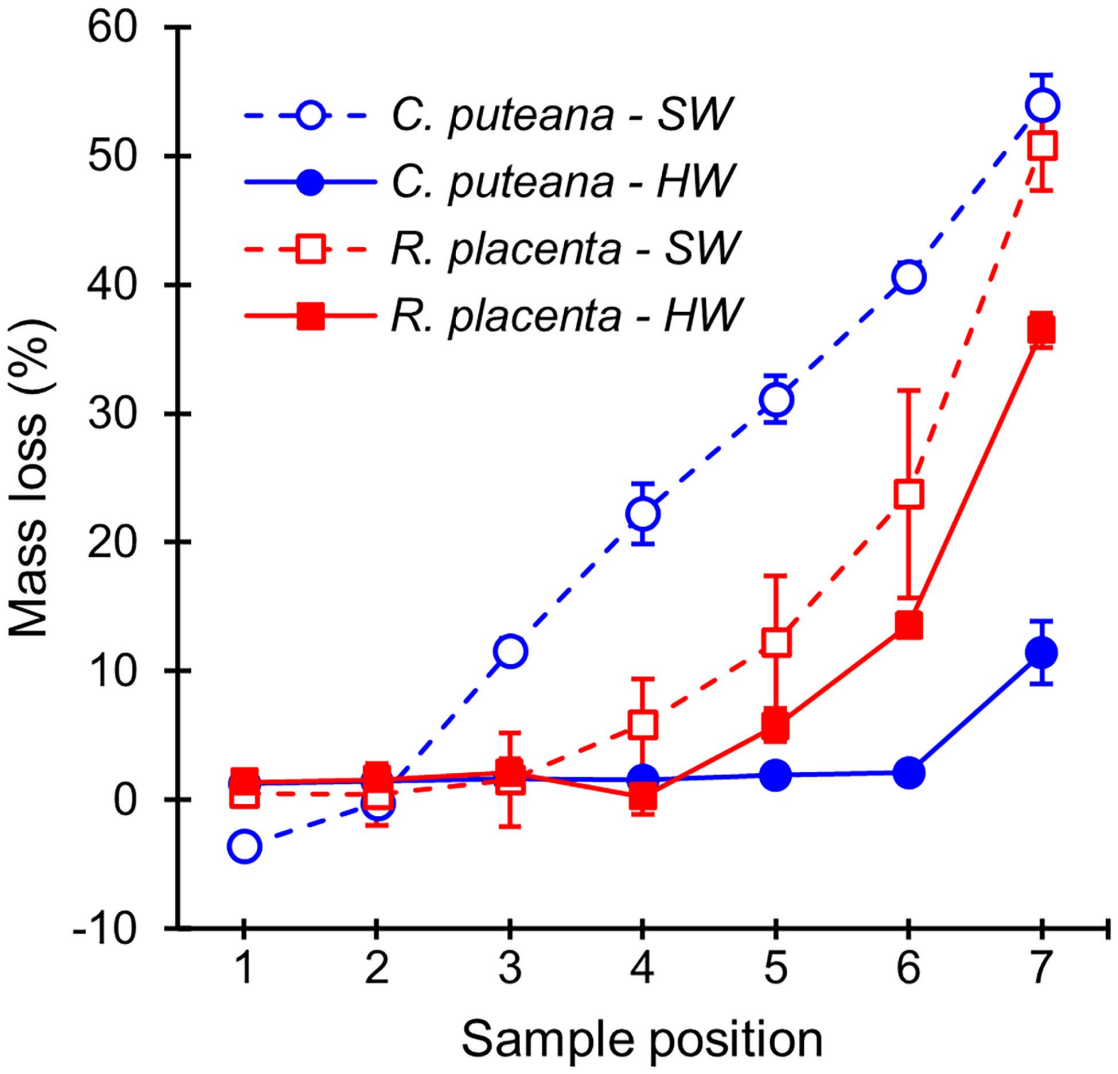
Average mass losses of Scots pine sapwood (SW) and heartwood (HW) blocks due to *C. puteana* and *R. placenta*. Error bars represent ±SD. *N* = 5.

The stacked wood sample decay test succeeded in producing a series of samples in different stages of decay (Figure 2). In the case of *C. puteana*, the sapwood samples showed a linear increase in mass loss from sample position 1 to position 7, reaching an average mass loss of 54.0% in the bottommost block (position 7). *Rhodonia placenta* produced a non-linear increase in sapwood mass loss, and apart from the bottommost block, the mass losses of the *R. placenta* degraded sapwood samples were substantially lower than their *C. puteana*-degraded equivalents. The heartwood samples were found to have substantial decay resistance against *C. puteana* but only limited resistance against *R. placenta*. In the case of *C. puteana*, only three of the seven heartwood blocks contained visible fungal mycelium (as opposed to all seven sapwood blocks), and only the bottommost block was degraded enough to show substantial mass loss (11.4%). By contrast, up to 6 of the 7 heartwood blocks contained visible mycelium in the case of *R. placenta*, and three of the bottommost samples had lost more than 5% of mass (5.8% at sample position 5 and 36.4% at sample position 7).

#### Degradation of Heartwood Extractives

**Figure 3:**
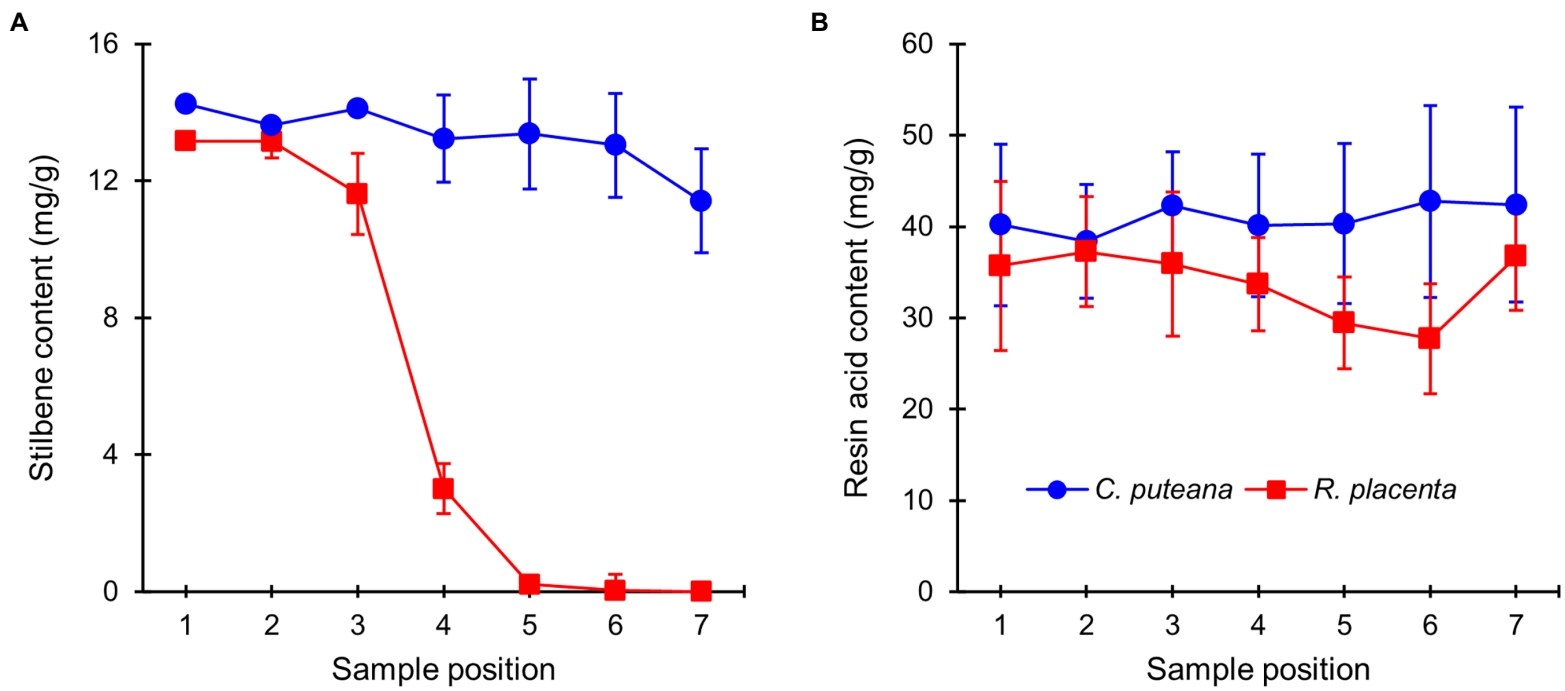
Average contents (mg/g) of initial undegraded stilbenes **(A)** and resin acids **(B)** in heartwood blocks exposed to *C. puteana* and *R. placenta*. Error bars represent ±SD. *N* = 5.

The extractive contents of the heartwood blocks were measured after the conclusion of the decay test. The total concentrations of the initial undegraded heartwood resin acids and stilbenes are given in Figure 3, while the concentrations of the individual compounds can be found in Supplementary Tables S1, S2 (the values in Supplementary Table S2 have been corrected for mass loss). Exposure to *C. puteana* had no statistically significant effect on the stilbene content of the samples, although a small decrease from 14.2 to 11.4 mg/g could be seen from the topmost (position 1) to the bottommost samples (position 7). In contrast to *C. puteana*, the samples exposed to *R. placenta* showed a rapid and extensive degradation of stilbenes. At a barely detectable average mass loss of 0.2% (position 4), the total stilbene content had decreased to 3.0 mg/g, while at 5.8% mass loss (position 5), the remaining stilbene content was only 0.2 mg/g. Both pinosylvin (PS) and pinosylvin monomethyl ether (PSM) were rapidly degraded. Resin acid content remained effectively unchanged in the samples exposed to *C. puteana*, while a small decrease from 35.1 to 27.7 mg/g could be seen from sample position 1 to position 6 in the *R. placenta*-degraded samples. However, due to variations in resin acid content between the replicate tubes, the differences were not statistically significant. Some differences in the extent of degradation could be observed between the individual resin acids, with neoabietic acid and other abietane resin acids showing the greatest decrease (Supplementary Table S2).

**Figure 4:**
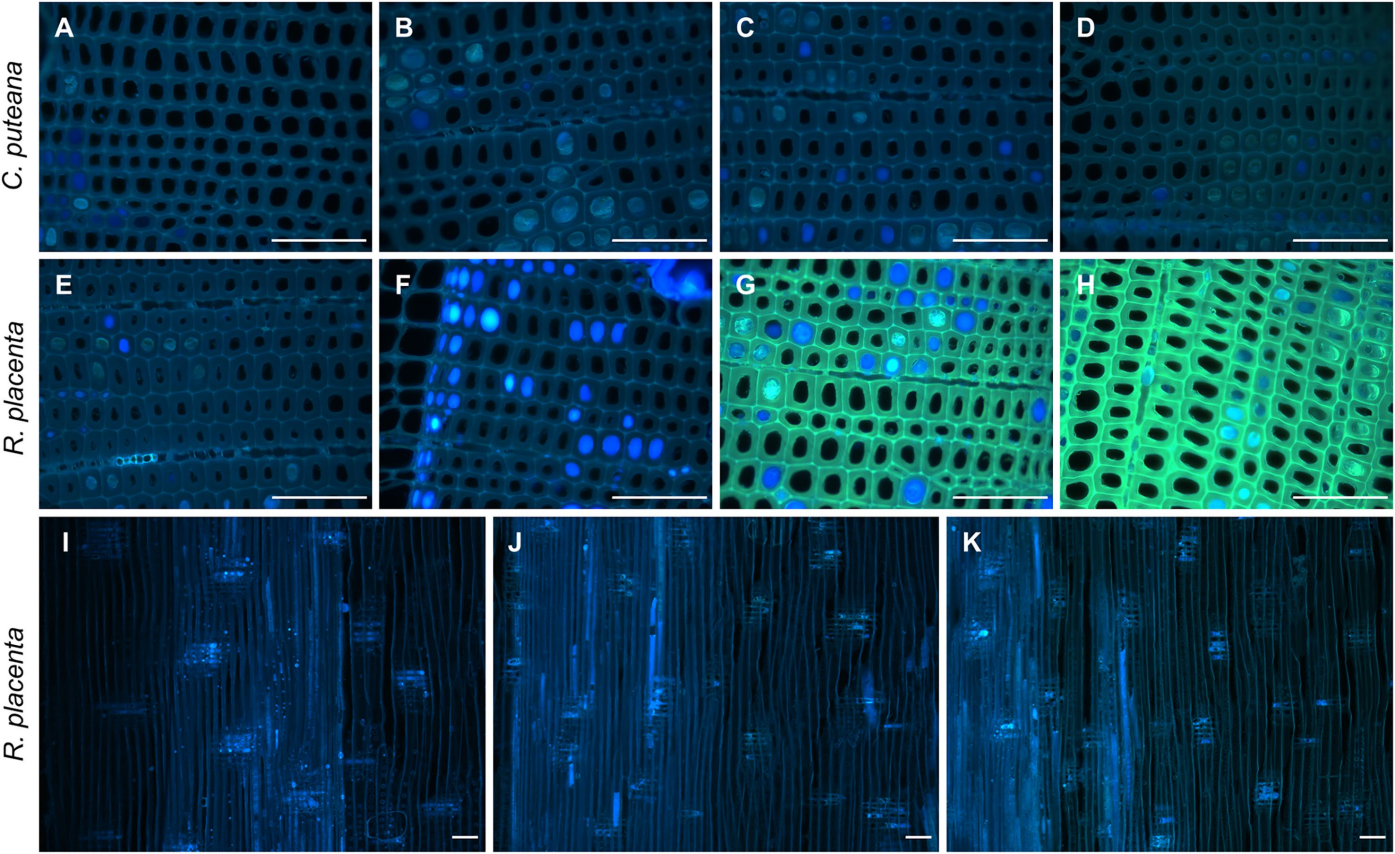
Fluorescence images (ex: 330–385 nm, em: 420-nm) from decayed heartwood blocks. Transverse sections from *C. puteana*-exposed wood at sample positions 1 **(A)**, 5 **(B)**, 6 **(C)**, and 7 **(D)**, transverse sections from *R. placenta*-exposed wood at positions 1 **(E)**, 3 **(F)**, 4 **(G)**, and 5 **(H)**, and radial sections from *R. placenta*-exposed wood at positions 1 **(I)**, 6 **(J)**, and 7 **(K)**. Images **(A–H)** were collected after 30 s of UV exposure in the microscope (330–385 nm), using the same acquisition settings for every image. Images **(I–K)** were collected without additional UV exposure. Scale bars are 0.1 mm.

On a microscopic level, the extractives of Scots pine heartwood are found in tracheid cell walls and in cell lumens as small deposits and large occlusions (Belt et al., 2017, 2020). Fluorescence microscopy was used to investigate the degradation of heartwood extractives on a cellular level. Under UV excitation, the cell walls of undegraded heartwood produced greyish blue fluorescence, while the extractive deposits fluoresced in different shades of blue and grey (Figure 4; Supplementary Figure S2). As could be expected based on the lack of extractive degradation (Figure 3), exposure to *C. puteana* had no effect on the distribution or fluorescence of heartwood extractives. In the case of *R. placenta*, no substantial changes could be seen in the sample at position 3, but at positions 4 and 5, the fluorescence characteristics of the material changed. The fluorescence of degraded heartwood became highly unstable, rapidly increasing in intensity and changing from blue to green when viewed under UV light in the fluorescence microscope. The fluorescence changes were present throughout the samples and affected all tracheid cell walls regardless of the presence of extractive deposits. The deposits developed greenish fluorescence as well, although many remained blue in samples at position 4 and 5. Apart from colour changes, no degradation of deposits (removal of material) could be detected, and images collected from radial sections confirmed that the deposits remained even in the most extensively degraded samples at positions 6 and 7. Localised areas of greenish fluorescence could sometimes be seen around rays in sapwood attacked by *C. puteana*, but otherwise no fluorescence changes were detected in *C. puteana* or *R. placenta*-degraded sapwood (Supplementary Figure S3).

#### Extractive Degradation Products in Decayed Heartwood Blocks

**Figure 5:**
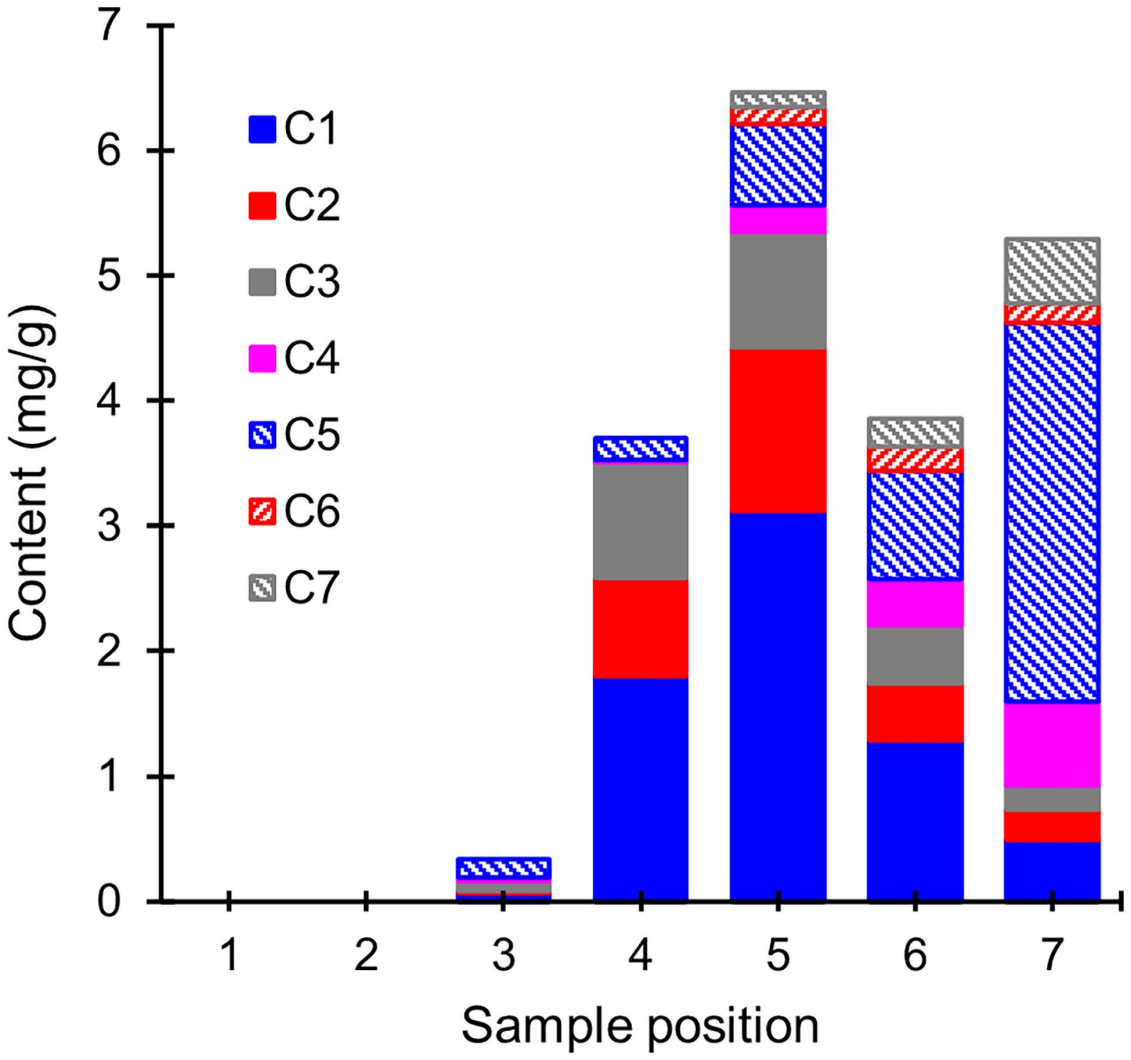
Average contents (mg/g) of extractive degradation products (compounds C1–C7) in heartwood blocks exposed to *R. placenta*. Compounds C1–C3 were identified as hydroxylated stilbene derivatives, and compounds C4–C7 as tentative hydroxylated dehydroabietic acid derivatives. *N* = 5.

To gain insight into the mechanisms behind extractive degradation, the extracts from heartwood blocks exposed to *R. placenta* were searched for potential extractive degradation products. In addition to several sugars, the GC–MS chromatograms of the degraded samples contained seven compounds (C1–C7) that were either absent in the undegraded samples or present in much lower concentrations (see Supplementary Figure S4). The concentrations of these compounds are given in Figure 5. Compounds C1–C3 were identified as hydroxylated derivatives of PS and PSM, while compounds C4–C7 were in turn tentatively identified as hydroxylated derivatives of dehydroabietic acid (DHA). The mass spectra of compounds C1 and C2 corresponded with resveratrol (PS + OH) and piceatannol (PS + 2OH), respectively, while compound C3 was tentatively identified as a di-hydroxylated derivative of PSM (Supplementary Figure S5). Only trace amounts of a compound identified as pinostilbene (PSM + OH) were detected in some samples. Compounds C4–C7 could not be conclusively identified, but their mass spectra (Supplementary Figure S6) shared many features in common with other mono-hydroxylated (C4) and di-hydroxylated (C5–C7) DHA derivatives.

The hydroxylated stilbene derivatives (C1–C3) first appeared at sample position 3 and increased in concentration until position 5. Comparisons of the initial PS and PSM contents (Supplementary Table S2) with the degradation product concentrations showed that most of the initial PS content (6.2 mg/g) was accounted for by the mixture of PS, resveratrol, and piceatannol at positions 4 and 5 (3.5 and 4.5 mg/g, respectively), while a smaller portion of the initial PSM content (7.0 mg/g) was found as a mixture of PSM and di-hydroxylated PSM (3.0 and 1.1 mg/g at positions 4 and 5, respectively). The concentration of hydroxylated stilbene derivatives declined after position 5, but no new stilbene derivatives detectable by GC–MS appeared in the extracts. Compound C5 appeared at position 3, while the other hydroxylated DHA derivatives appeared at position 5. The abundance of the DHA derivatives increased towards position 7, at which point their concentration exceeded the amount of DHA lost from the wood material (Supplementary Table S2).

### Extractive Degradation Assays

**Figure 6:**
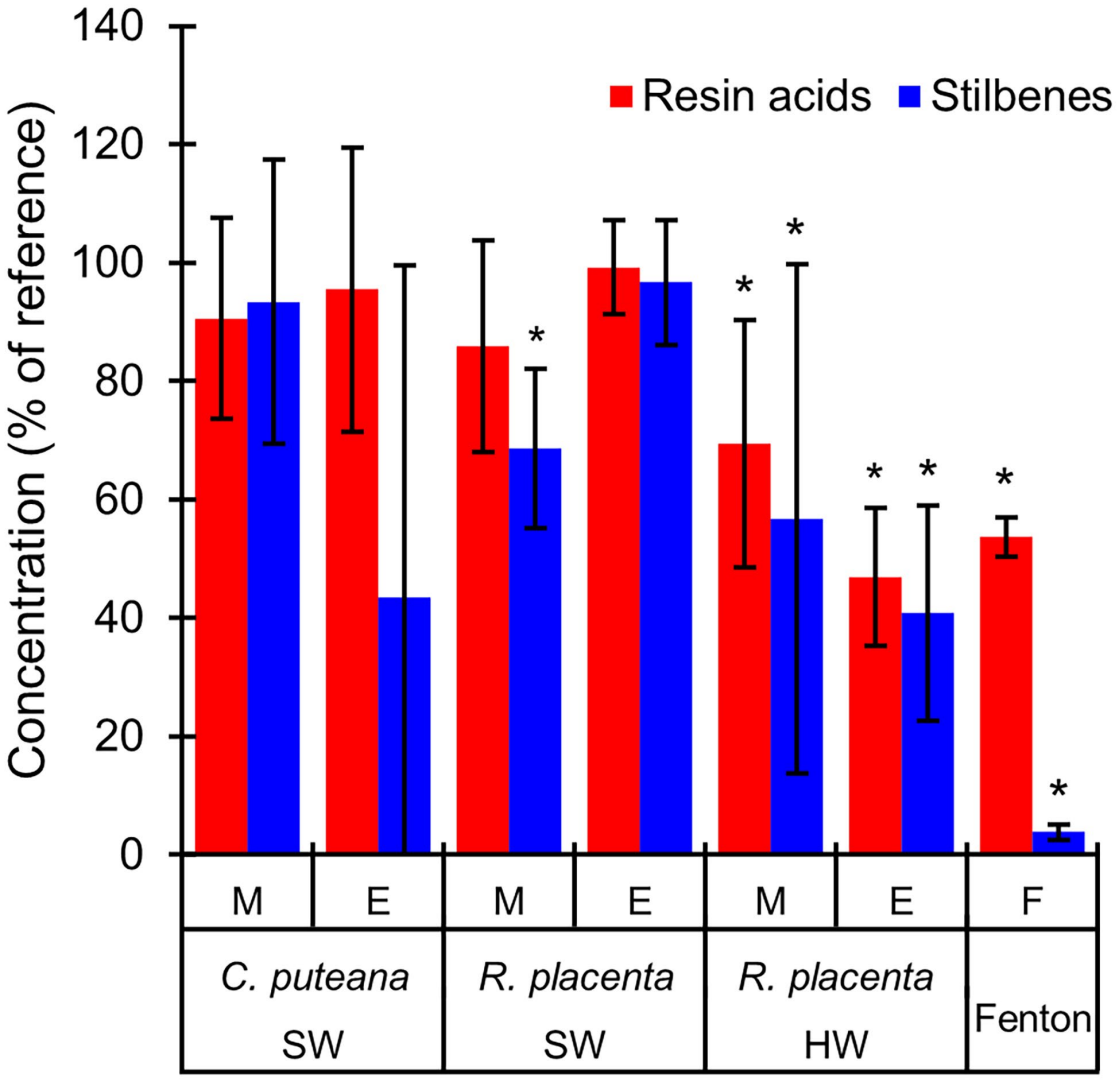
Average concentrations (as % of reference) of the original stilbenes and resin acids in the heartwood extract after incubation with mycelium (M) or extracellular culture fluid (E) from *C. puteana* and *R. placenta* cultures supplemented with sapwood (SW) or heartwood (HW) powder, or with Fenton reagent. *Coniophora puteana* did not grow in heartwood-supplemented culture. Error bars represent ±SD. *Statistically significant difference to reference (*p* < 0.05 according to Student’s *t*-test). *N* = 5.

The involvement of the different fungal degradative systems in extractive degradation was investigated using simple degradation assays. A methanolic extract obtained from Scots pine heartwood was incubated for 6 h with fungal mycelium or extracellular culture fluid (from liquid *C. puteana* and *R. placenta* cultures supplemented with sapwood or heartwood powder) or with neat Fenton reagent. Changes were assessed by comparing extractive concentrations after incubation to a suitable reference incubated for the same duration (mycelium was compared to autoclaved mycelium, extracellular culture fluid to fresh malt extract broth, and the Fenton reaction to plain buffer). The changes in the total concentration of the initial undegraded stilbenes and resin acids are given in Figure 6, while the changes in the concentrations of the individual compounds are given in Table 1.

**Table 1:** Average concentrations (as % of reference) of individual stilbenes and resin acids in the heartwood extract after incubation with mycelium (M) or extracellular culture fluid (E) from *C. puteana* and *R. placenta* cultures supplemented with sapwood (SW) or heartwood (HW) powder, or with Fenton reagent.

	*C. puteana*—SW		*R. placenta*—SW		*R. placenta*—HW		Fenton
	*M*	*E*	*M*	*E*	*M*	*E*	*F*
PS	94.9 (18.9)	42.0 (54.3)	73.9 (13.1)	92.5 (9.8)	65.9 (52.8)	34.0 (6.8)	0 (0.0)
PSM	92.1 (19.2)	44.8 (57.7)	63.6 (13.9)	100.4 (11.7)	47.4 (64.6)	41.5 (8.4)	7.4 (2.5)
PiA	91.0 (19.4)	105.1 (10.5)	85.3 (14.7)	99.0 (5.3)	69.0 (24.9)	40.5 (27.9)	92.7 (5.9)
SPiA	89.3 (34.6)	110.9 (33.8)	106.2 (40.1)	108.9 (21.4)	67.2 (43.6)	60.8 (15.0)	88.2 (16.0)
IPiA	88.2 (21.6)	106.0 (14.6)	91.5 (16.4)	90.8 (18.7)	74.0 (31.6)	40.0 (7.7)	86.6 (10.6)
PA	91.7 (18.4)	80.5 (39.5)	78.0 (13.8)	98.0 (7.4)	75.3 (24.2)	50.3 (7.6)	43.5 (6.3)
LPiA	94.0 (18.4)	43.4 (56.5)	82.9 (14.9)	84.5 (9.3)	69.8 (34.0)	76.9 (11.5)	19.4 (1.5)
DHA	91.8 (25.4)	111.7 (11.6)	84.6 (20.3)	107.9 (12.6)	64.6 (22.5)	13.0 (7.2)	112.4 (8.3)
AA	87.7 (18.3)	151.7 (46.5)	87.8 (14.2)	110.5 (7.4)	70.6 (21.5)	40.4 (7.7)	36.3 (3.8)
NAA	89.0 (22.9)	63.5 (42.9)	84.6 (15.5)	99.6 (9.8)	66.8 (40.7)	56.2 (7.1)	18.5 (2.7)

*PS, pinosylvin; PSM, pinosylvin monomethyl ether; PiA, pimaric acid; SPiA, sandaracopimaric acid; IPiA, isopimaric acid, PA, palustric acid; LPiA, levopimaric acid; DHA, dehydroabietic acid; AA, abietic acid; and NAA, neoabietic acid*.

*Coniophora puteana did not grow in heartwood-supplemented culture. Values in parentheses are ±SD. *N* = 5*.

Incubation of the heartwood extract with *C. puteana* mycelium from the sapwood-supplemented culture caused no changes in stilbene and resin acid content, while incubation with the extracellular fluid produced changes in both stilbenes and resin acids but only in 3 out of 5 replicate cultures. The cultures showing changes contained no PS or PSM at the end of the incubation period, and although there was little change in the total concentration of resin acids, they contained no levopimaric acid and showed reductions in the concentration of palustric and neoabietic acid and increases in the concentration of abietic acid. The effects of heartwood on extractive degradation could not be tested with *C. puteana* because the fungus failed to grow in the heartwood-supplemented culture.

Incubation of the heartwood extract with *R. placenta* mycelium from the sapwood-supplemented culture resulted in a 31% decrease in stilbene content and a statistically insignificant decrease in resin acid content. No changes in resin acid or stilbene content were recorded after incubation with extracellular fluid. The mycelium originating from heartwood-supplemented *R. placenta* cultures reduced the concentrations of stilbenes and resin acids to 53 and 69% of the reference, respectively, while the extracellular fluid reduced stilbene and resin acid concentrations to 41 and 47% of the relevant reference. Individual resin acids and stilbenes were generally degraded to similar extents in all *R. placenta* mycelium incubations, while some differences between the individual compounds could be seen with extracellular fluid derived from the heartwood-supplemented culture. DHA in particular was extensively degraded by the extracellular fluid.

The Fenton reaction proved highly efficient at degrading heartwood extractives at the reagent concentrations applied in this experiment. The total concentration of the initial stilbenes was reduced to 4% of the reference, while the total concentration of resin acids decreased to 44%. There were large differences in degradation between the different resin acids: the concentration of the non-aromatic abietane resin acids was reduced to 19%–44% of the reference, while the concentration of the pimarane resin acids was only reduced to 87%–93%. The concentration of DHA increased slightly.

**Figure 7:**
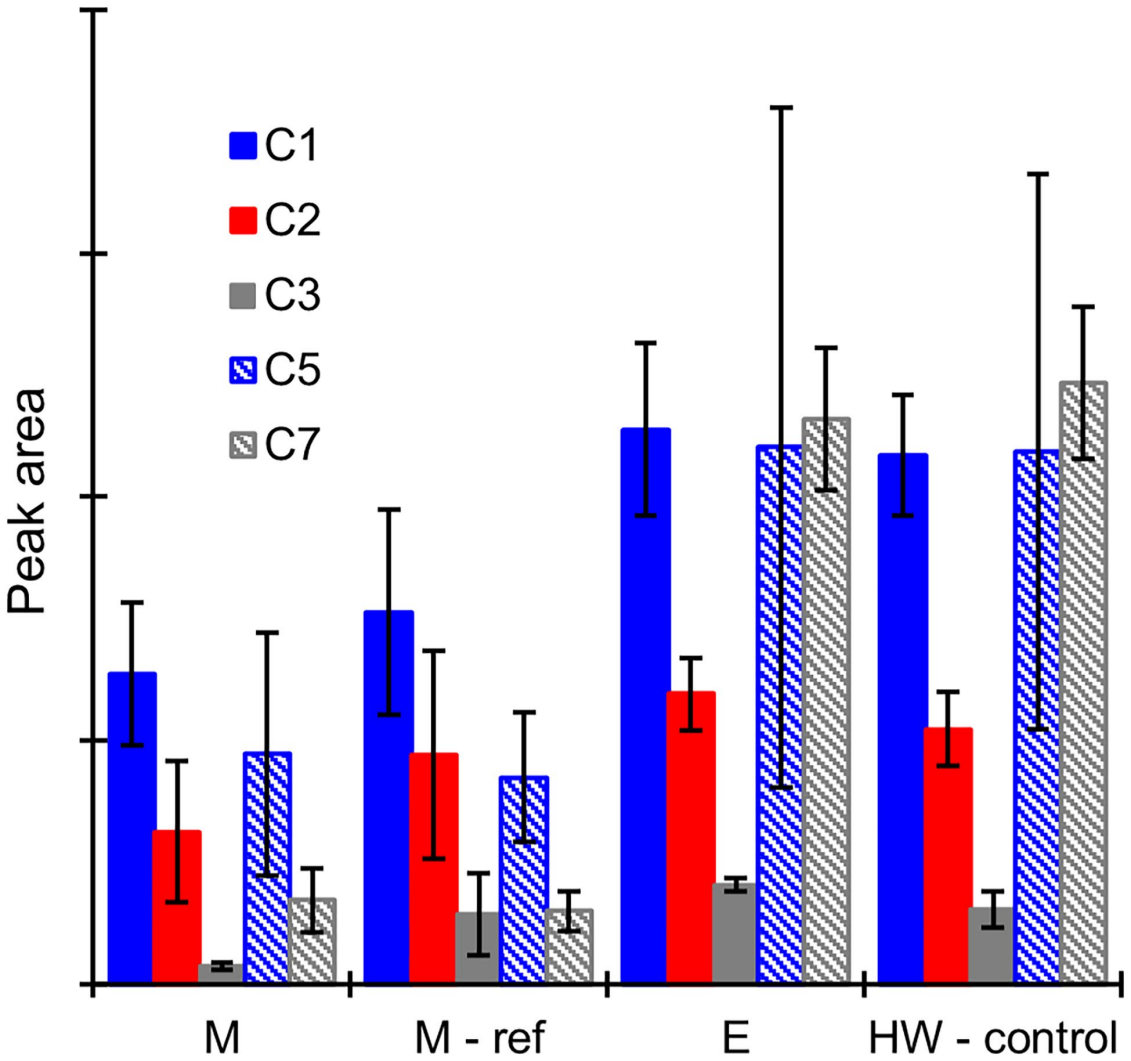
Average GC–MS peak areas of degradation products C1, C2, C3, C5, and C7 in the heartwood extract after incubation with mycelium (M), autoclaved mycelium (M-ref), and extracellular culture fluid (E) derived from heartwood-supplemented *R. placenta* cultures, and in the control sample of extracellular culture fluid (HW-control) analysed without incubation with the heartwood extract. Error bars represent ±SD. No differences between M and M-ref and between E and HW-control were statistically significant (*p* > 0.05 according to Student’s *t*-test). *N* = 5.

No potential extractive degradation products were found on the GC–MS chromatograms when the heartwood extract was incubated with mycelium or extracellular fluid from the sapwood-supplemented *C. puteana* or *R. placenta* cultures (see Supplementary Figure S7). Several small peaks were detected when the extract was incubated with Fenton reagent (see Supplementary Figure S8), but none of the compounds matched the compounds found in the *R. placenta*-degraded heartwood blocks. Several hydroxylated degradation products were present in the incubations involving mycelium or extracellular fluid derived from the heartwood-supplemented *R. placenta* cultures, and these compounds (along with large concentrations of undegraded stilbenes and resin acids) were also present in the control sample of culture fluid that was analysed without incubation with the heartwood extract. The peak areas of the degradation products after incubation of the heartwood extract with mycelium, autoclaved mycelium (used as the reference for mycelium incubations), or extracellular fluid are given in Figure 7, along with the peak areas of the degradation products in the control sample of extracellular fluid. Substantial quantities of degradation products were found after incubation with the mycelium and autoclaved mycelium even though the incubations had been carried out in fresh malt extract. The concentrations of compounds C1 (PS + OH), C2 (PS + 2OH), and C3 (PSM + 2OH) were particularly high and comparable to the heartwood culture fluid. The concentrations of compounds C1–C3 were lower in the live than autoclaved mycelium incubations, but the differences were not statistically significant. The concentrations of compounds C5 and C7 showed no significant difference between the live and autoclaved mycelium incubations. The extracellular fluid incubation and the control sample of extracellular fluid contained similar concentrations of degradation products, which shows that 6-h incubation period had no effect on degradation product concentrations in the extracellular fluid.

## Discussion

Mass losses measured after the stacked-sample decay test (Figure 2) showed that the heartwood of Scots pine had substantial decay resistance against *C. puteana* but little resistance against *R. placenta*. We hypothesised that the differences in heartwood degrading efficiency between the two fungi were due to differences in their ability to degrade the antifungal heartwood extractives. Previous research has shown that both fungal species can degrade heartwood stilbenes (Karppanen et al., 2008; Lu et al., 2016; Belt et al., 2019), but our extractive content measurements demonstrated that the stilbene-degrading efficiency of *R. placenta* was much higher than that of *C. puteana*: at less than 1% mass loss, the stilbene content of the *R. placenta*-exposed blocks had decreased to less than 25% of the original, while the stilbene content of the *C. puteana*-exposed blocks showed only a slight decrease at 11% mass loss (Figure 3). *Rhodonia placenta* also caused a noticeable but statistically insignificant decrease in total resin acid content (Figure 3). The differences in the rate of degradation between stilbenes and resin acids are most likely a reflection of differences in their antifungal activity. Stilbenes are much more antifungal than resin acids (Wijayanto et al., 2015), although it is possible that their higher antioxidant activity also makes them more prone to oxidative degradation than the resin acids.

On a microscopic level, the Scots pine heartwood stilbenes are found in tracheid cell walls, and in cell lumens as small deposits and large occlusions in combination with resin acids (Belt et al., 2017, 2020). Fluorescence microscopy (Figure 4) showed that *R. placenta* degradation changed the colour and intensity of heartwood cell wall fluorescence, but there were no signs of deposit degradation (removal of material) even in advanced decay. The persistence of the lumen-filling deposits shows that their physical blocking action is not sufficient to hinder the degradative action of *R. placenta*. The fluorescence changes were present throughout the heartwood material and affected all tracheid cell walls regardless of the presence of extractive deposits, which suggests that the degradation of extractives proceeds through the same diffusible mechanism as cell wall degradation.

Analysis of the extracts from the *R. placenta*-degraded heartwood blocks (Figure 5) revealed the presence of hydroxylated stilbene and resin acid derivatives, which have not been previously identified in natural heartwood decayed by brown rot fungi. Hydroxylated derivatives of PS and PSM have been detected in brown rot decayed wood impregnated with a solution of heartwood stilbenes (Lu et al., 2016), while hydroxylated derivatives of DHA have been found in biotransformation experiments of abietic acid and other abietane resin acids using different types of filamentous fungi (Özşen et al., 2017; Luchnikova et al., 2019). In this study, the accumulation pattern of the stilbene derivatives mirrored the pattern of initial stilbene degradation until position 5, after which their concentration decreased and no new stilbene derivatives were detected. The hydroxylated resin acid derivatives accumulated in the later stages of decay, in agreement with their slower rate of degradation. All the detected compounds were derivatives of DHA, which suggests that the degradation of resin acids by *R. placenta* proceeds through a DHA intermediate.

The extract degradation assays (Figure 6; Table 1) showed that the degradation/elimination of extractives could be achieved by both fungal mycelium and extracellular culture fluid. However, differences were seen between the two fungi, which suggest that the intracellular detoxification system plays an important role in the resistance to heartwood extractives. *Rhodonia placenta* was able to eliminate extractives using both its mycelium and extracellular culture fluid when grown in heartwood-supplemented culture, while *C. puteana* did not grow at all in heartwood-containing culture and was only able to eliminate extractives using its extracellular fluid when grown in sapwood-supplemented culture. The degradation of extractives by the mycelium can involve cytochrome P450 monooxygenases and transferases (Morel et al., 2013); the P450s are known to catalyse the hydroxylation of various compounds (Durairaj et al., 2016) and may thus be responsible for the hydroxylated degradation products found in the *R. placenta*-degraded wood blocks. *Rhodonia placenta* has the largest complement of P450s among the sequenced wood decaying fungi, with 345 genes encoding for P450s compared to *C. puteana*’s 224 (Park et al., 2008). However, there were differences in the degradation patterns of the individual extractive compounds between *R. placenta* mycelium and *R. placenta* wood decay (Figure 3; Supplementary Table S2), which suggest that other degradative mechanisms are also involved in extractive degradation.

In contrast to the intracellular detoxification system, the significance of the extracellular enzyme activities in extractive degradation is difficult to assess. The extracellular fluid incubations did not produce any detectable degradation products, which mean that they are not responsible for the production of the hydroxylated degradation products seen in *R. placenta*-degraded heartwood. The activities involved remain unidentified and may in fact be different in *C. puteana* and *R. placenta*, as suggested by the differences in the degradation patterns of the individual resin acids (Table 1). Extracellular enzymes potentially involved in extractive degradation include various oxidoreductases such as laccases and peroxidases. Brown rot fungi do not produce the classical lignin-degrading peroxidases found in white rot fungi (lignin peroxidase, manganese-dependent peroxidase, and versatile peroxidase), but they can produce other types of peroxidases as well as laccases (Kersten and Cullen, 2014). Many oxidoreductases can catalyse the dimerization of stilbenes, but successful reaction requires the presence of a 4’-OH (Keylor et al., 2015), which the pine heartwood stilbenes lack. The activation of the meta-OH-groups found on PS and PSM produces an unstable radical that can deactivate enzymes (Szewczuk et al., 2004, 2005), and it is unclear what products result from this reaction. Oxidoreductases such as laccases can also degrade resin acids in the presence of suitable mediators (Gutiérrez et al., 2006). The lack of degradation products in the extracellular fluid incubations suggests that the extracellular enzymes generate products that are too unstable or too large for GC analysis.

In addition to the intracellular and extracellular enzymes, the Fenton reaction was also highly effective at degrading extractives. The hydroxyl radicals derived from the Fenton reaction can attack a variety of organic compounds, primarily by abstracting hydrogen atoms or by adding to unsaturated bonds (Pignatello et al., 2006). Hydroxylation is a typical outcome of hydroxyl radical attack on aromatic compounds, which means that the Fenton reaction may also contribute to the formation of hydroxylated extractive degradation products in addition to the P450s. *Rhodonia placenta* heartwood decay and the simple Fenton reaction utilised in the degradation assays produced similar patterns of extractive degradation characterised by extensive degradation of stilbenes and more pronounced degradation of abietane than pimarane resin acids, but no hydroxylated degradation products were detected when the heartwood extract was incubated with Fenton reagent (Supplementary Figure S8). However, this may have been due to the very high level of degradation in the assays and whatever differences may exist between the simple Fenton reaction in solution and the fungal chelator-mediated Fenton reaction in solid wood. The Fenton reaction is the first step in the brown rot degradative process and causes the depolymerisation of the cell wall carbohydrates, possibly resulting in the production of soluble fragments that diffuse out of the cell wall (Goodell et al., 2017). It is likely that the extractives are also attacked by the hydroxyl radicals forming within the cell walls during heartwood decay. The radical-attacked extractives may then remain in the cell walls, or they may diffuse out of the cell walls and be subjected to further fungal degradation.

Taken together, the results of the decay test and the extract degradation assays suggest that extractive degradation plays an important role in heartwood decay. Stilbene degradation appears to be particularly important, with rapid stilbene degradation in incipient decay emerging as a defining difference between *C. puteana* and *R. placenta*. The exact mechanisms of extractive degradation could not be identified, and it is possible that the degradation is in fact achieved through the overlapping action of several degradative systems. The hydroxylated degradation products seen in *R. placenta*-degraded heartwood may be produced by one or several degradative systems, but the degradation is likely to also proceed through other routes, as suggested by the fact that only a portion of the stilbenes and resin acids lost from the wood material could be recovered as their hydroxylated derivatives in any given sample. The extractives may be taken up by the mycelium and processed intracellularly without excretion, or they may be converted to products undetectable by GC by the extracellular enzymes or the Fenton reaction. The hydroxylated derivatives may also be taken up by the mycelium and/or processed further by the extracellular enzymes and the Fenton reaction, which would explain the disappearance of the hydroxylated stilbenes in advanced decay. Overall, the results highlight the complexity of the fungal degradative systems and the differences in degradative ability between fungal species. The degradative abilities of *R. placenta* have been previously demonstrated against a variety of decay resistant wood materials, including larch heartwood (Gierlinger et al., 2004), copper preservative treated wood (Sierra-Alvarez, 2007), and heat-treated wood (Kamdem et al., 2002), and its degradative systems warrant further investigation.

## Data Availability Statement

The raw data supporting the conclusions of this article will be made available by the authors, without undue reservation.

## Author Contributions

TB conceived and designed the study, performed the experiments, analysed the data in cooperation with the authors and wrote the manuscript with contributions from all authors. All authors contributed to the article and approved the submitted version.

## Funding

This work received funding from the Academy of Finland (grant no. 330087) and Jenny and Antti Wihuri foundation.

## Conflict of Interest

The authors declare that the research was conducted in the absence of any commercial or financial relationships that could be construed as a potential conflict of interest.

## Publisher’s Note

All claims expressed in this article are solely those of the authors and do not necessarily represent those of their affiliated organizations, or those of the publisher, the editors and the reviewers. Any product that may be evaluated in this article, or claim that may be made by its manufacturer, is not guaranteed or endorsed by the publisher.

## Supplementary Material

The Supplementary Material for this article can be found online at: https://www.frontiersin.org/articles/10.3389/fpls.2022.912555/full#supplementary-material
