# Supplementary material for: Fungal Degradation of Extractives Plays an Important Role in the Brown Rot Decay of Scots Pine Heartwood
Source: Front Plant Sci. 2022 May 12;13:912555. doi: 10.3389/fpls.2022.912555 (PMC9133955; doi:10.3389/fpls.2022.912555)
Supplement: Supplementary file 1 [file Data_Sheet_1.PDF]

Supplementary Material

Supplementary Table 1. Average content (mg/g) of the initial undegraded stilbenes and resin acids in heartwood blocks exposed to *C. puteana* and *R. placenta*.

Fungus	Pos.	PS	PSM	PiA	SPiA	IPiA	PA	LPiA	DHA	AA	NAA
	1	6.3	7.9	3.8	0.3	1.1	6.6	4.1	10.5	7.1	6.7
	2	6.0	7.6	3.6	0.3	1.1	6.4	3.8	10.1	6.8	6.3
	3	6.1	8.0	4.1	0.3	1.1	7.1	4.2	11.1	7.4	7.0
C. puteana	4	5.8	7.4	3.9	0.3	1.1	6.7	3.9	10.6	7.1	6.6
	5	5.9	7.5	3.8	0.3	1.1	6.7	4.1	10.5	7.2	6.7
	6	5.6	7.4	4.0	0.3	1.2	7.1	4.5	11.1	7.5	7.1
	7	4.6	6.8	3.8	0.3	1.2	7.0	3.8	11.7	7.8	6.8
	1	6.2	7.0	3.3	0.5	0.4	6.2	3.5	9.4	7.0	5.3
	2	6.1	7.1	3.7	0.2	0.8	6.8	3.9	10.5	7.1	4.4
	3	5.2	6.4	3.4	0.2	0.8	6.6	3.6	9.8	7.3	4.3
R. placenta	4	0.9	2.1	3.1	0.2	0.6	6.0	2.6	9.0	7.9	4.2
	5	0.0	0.2	3.0	0.2	0.5	5.2	2.6	8.5	6.3	3.2
	6	0.0	0.0	2.9	0.2	0.5	4.7	2.2	8.4	6.1	2.7
	7	0.0	0.0	4.1	0.3	0.7	6.2	2.9	11.1	7.8	3.6

PS, pinosylvin; PSM, pinosylvin monomethyl ether; PiA, pimaric acid; SPiA, sandaracopimaric acid; IPiA, isopimaric acid, PA, palustric acid; LPiA, levopimaric acid; DHA, dehydroabietic acid; AA, abietic acid; NAA, neoabietic acid

Supplementary Table 2. Average content (mg/g) of the initial undegraded stilbenes and resin acids in heartwood blocks exposed to *C. puteana* and *R. placenta* adjusted for mass loss

Fungus	Pos.	PS	PSM	PiA	SPiA	IPiA	PA	LPiA	DHA	AA	NAA
	1	6.3	7.9	3.8	0.3	1.1	6.6	4.1	10.5	7.1	6.7
	2	6.0	7.6	3.6	0.3	1.1	6.4	3.8	10.1	6.8	6.3
	3	6.1	8.0	4.1	0.3	1.1	7.1	4.2	11.1	7.4	7.0
C. puteana	4	5.8	7.4	3.9	0.3	1.1	6.7	3.9	10.6	7.1	6.6
	5	5.9	7.5	3.8	0.3	1.1	6.7	4.1	10.5	7.2	6.7
	6	5.6	7.4	4.0	0.3	1.2	7.1	4.5	11.1	7.5	7.1
	7	4.6	6.8	3.8	0.3	1.2	7.0	3.8	11.7	7.8	6.8
	1	6.2	7.0	3.3	0.5	0.4	6.2	3.5	9.4	7.0	5.3
	2	6.1	7.1	3.7	0.2	0.8	6.8	3.9	10.5	7.1	4.4
	3	5.2	6.4	3.4	0.2	0.8	6.6	3.6	9.8	7.3	4.3
R. placenta	4	0.9	2.1	3.1	0.2	0.6	6.0	2.6	9.0	7.9	4.2
	5	0.0	0.2	3.0	0.2	0.5	5.2	2.6	8.5	6.3	3.2
	6	0.0	0.0	2.9	0.2	0.5	4.7	2.2	8.4	6.1	2.7
	7	0.0	0.0	4.1	0.3	0.7	6.2	2.9	11.1	7.8	3.6

PS, pinosylvin; PSM, pinosylvin monomethyl ether; PiA, pimaric acid; SPiA, sandaracopimaric acid; IPiA, isopimaric acid, PA, palustric acid; LPiA, levopimaric acid; DHA, dehydroabietic acid; AA, abietic acid; NAA, neoabietic acid

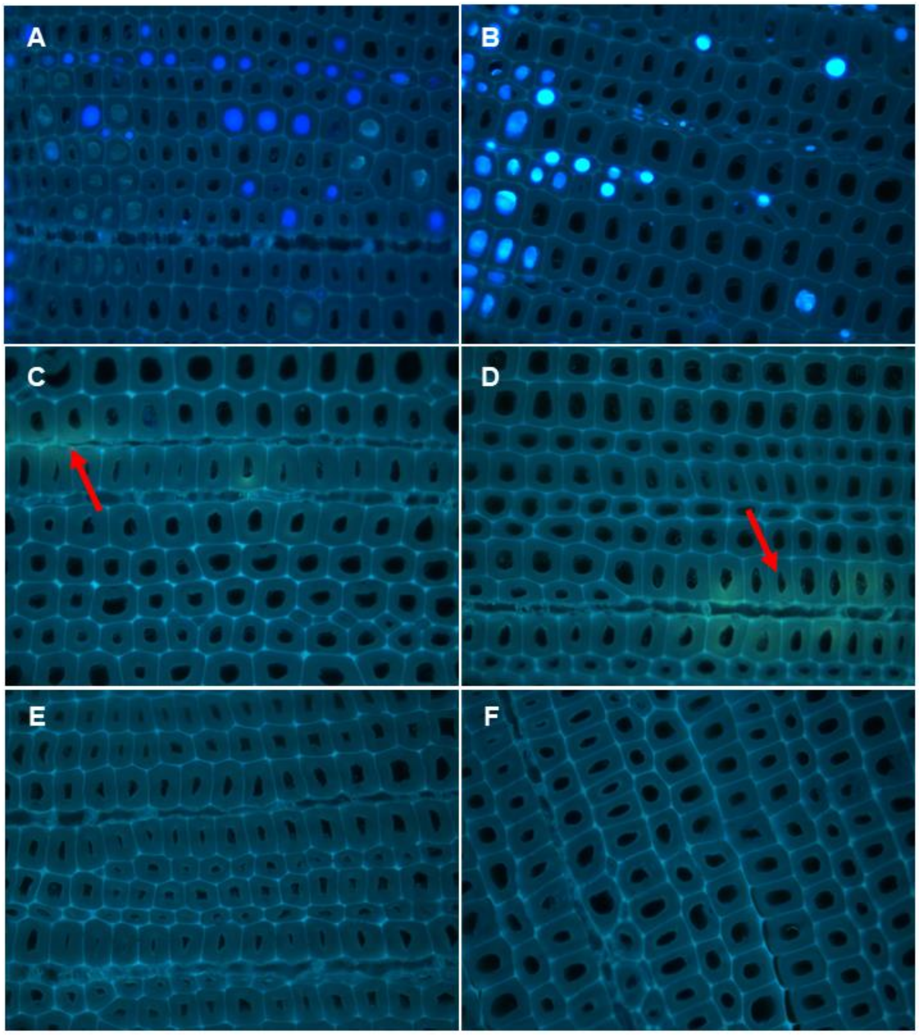

Supplementary Figure 3. Fluorescence images (ex: 330–385 nm, em: 420- nm) from undegraded heartwood showing deposits fluorescing in different colours (**A**, **B**), from *C. puteana*-degraded sapwood at sample positions 1 (**C**) and 2 (**D**), and from *R. placenta*-degraded sapwood at sample positions 2 (**E**) and 4 (**F**). Red arrows in (**C**) and (**D**) point to areas of altered fluorescence colour. Different exposure times were used for the heartwood and sapwood samples

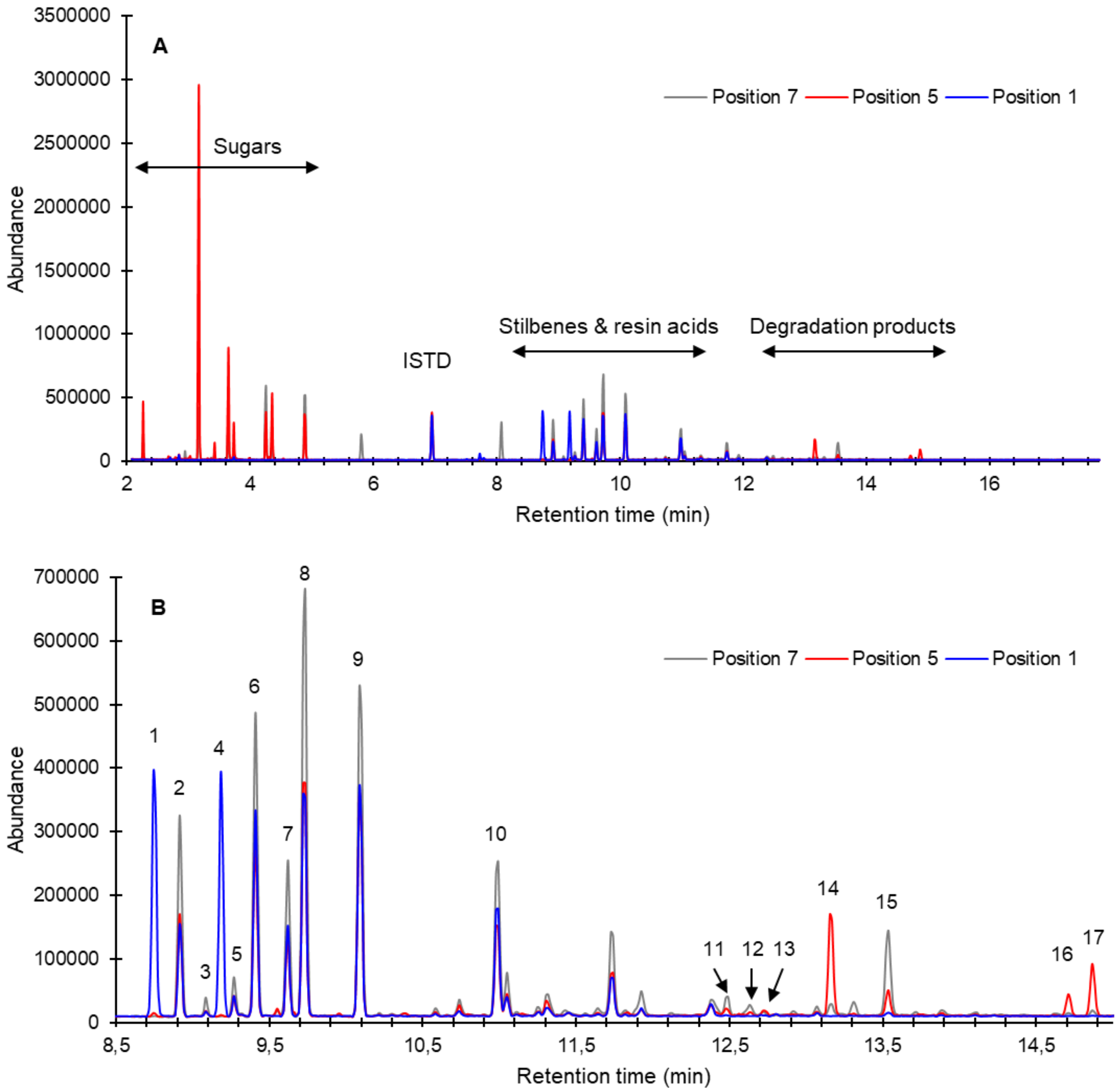

Supplementary Figure 4. Example GC-MS total ion chromatograms of heartwood samples at positions 1, 5 and 7 from 2 to 17.8 min (**A**) and from 8.5 to 15 min (**B**). Peak identities in (**B**): pinosylvin monomethyl ether (1), pimaric acid (2), sandaracopimaric acid (3), pinosylvin (4), isopimaric acid (5), palustric acid (6), levopimaric acid (7), dehydroabietic acid (8), abietic acid (9), neoabietic acid (10), C4 (11), C7 (12), C6 (13), C1 (14), C5 (15), C3 (16), C2 (17)

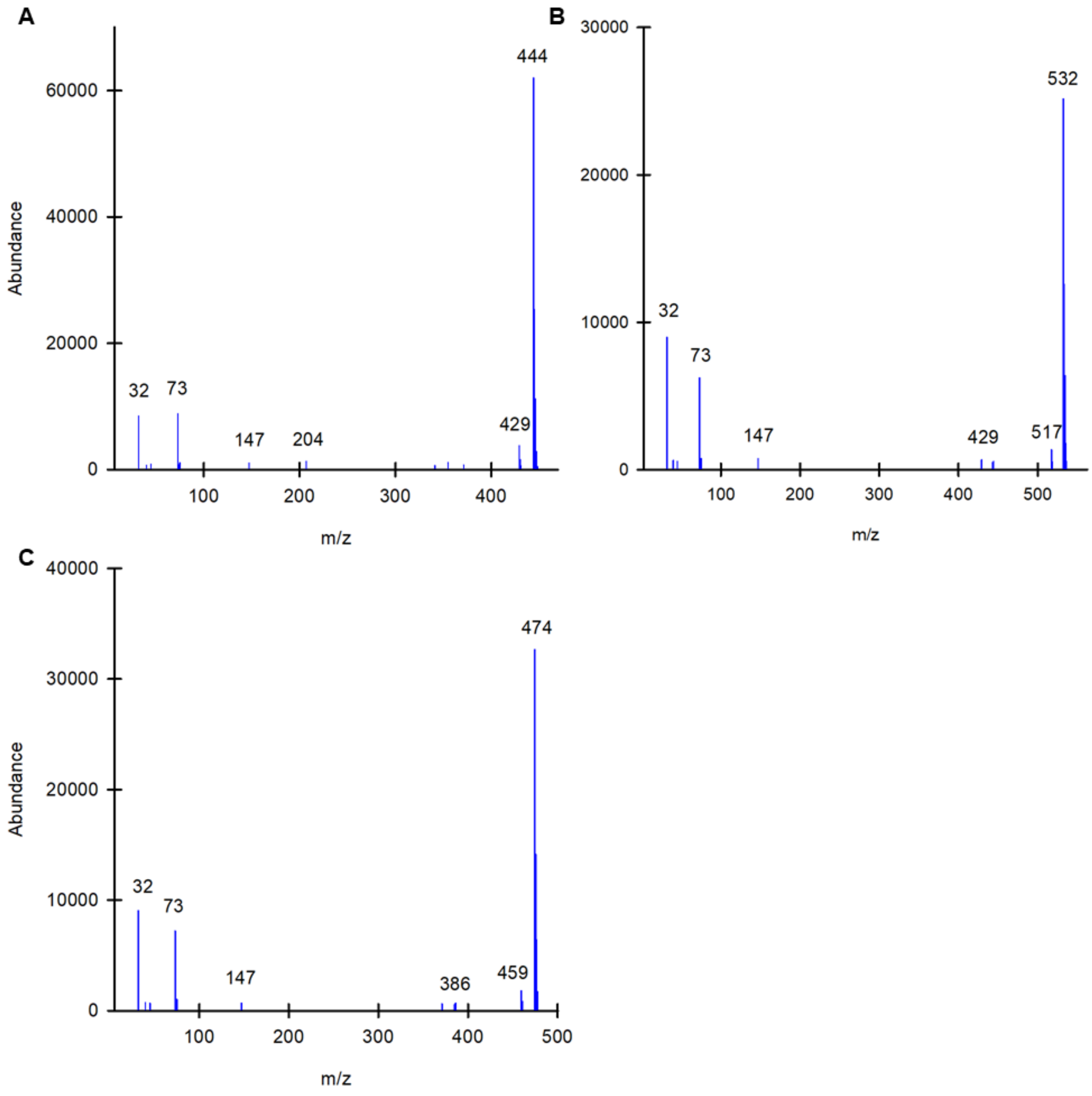

Supplementary Figure 5. Mass spectra of degradation products C1-C3. Compounds C1 (**A**) and C2 (**B**) were identified as resveratrol and piceatannol, respectively, while C3 (**C**) was tentatively identified as di-hydroxylated pinosylvin monomethyl ether

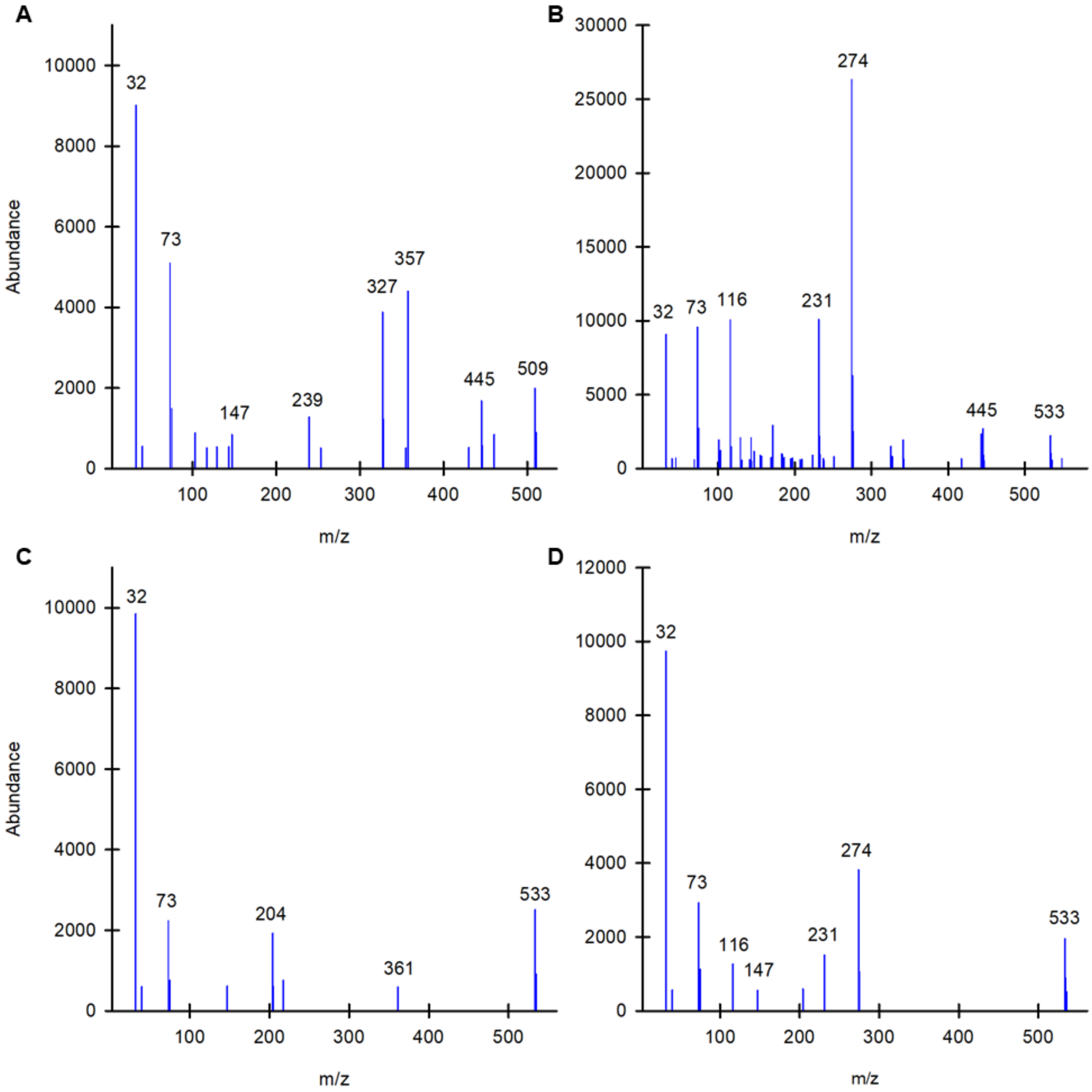

Supplementary Figure 6. Mass spectra of degradation products C4-C7. Compound C4 (**A**) was tentatively identified as a mono-hydroxylated derivative of dehydroabietic acid, while compounds C5 (**B**), C6 (**C**) and C7 (**D**) were tentatively identified as di-hydroxylated derivatives of dehydroabietic acid

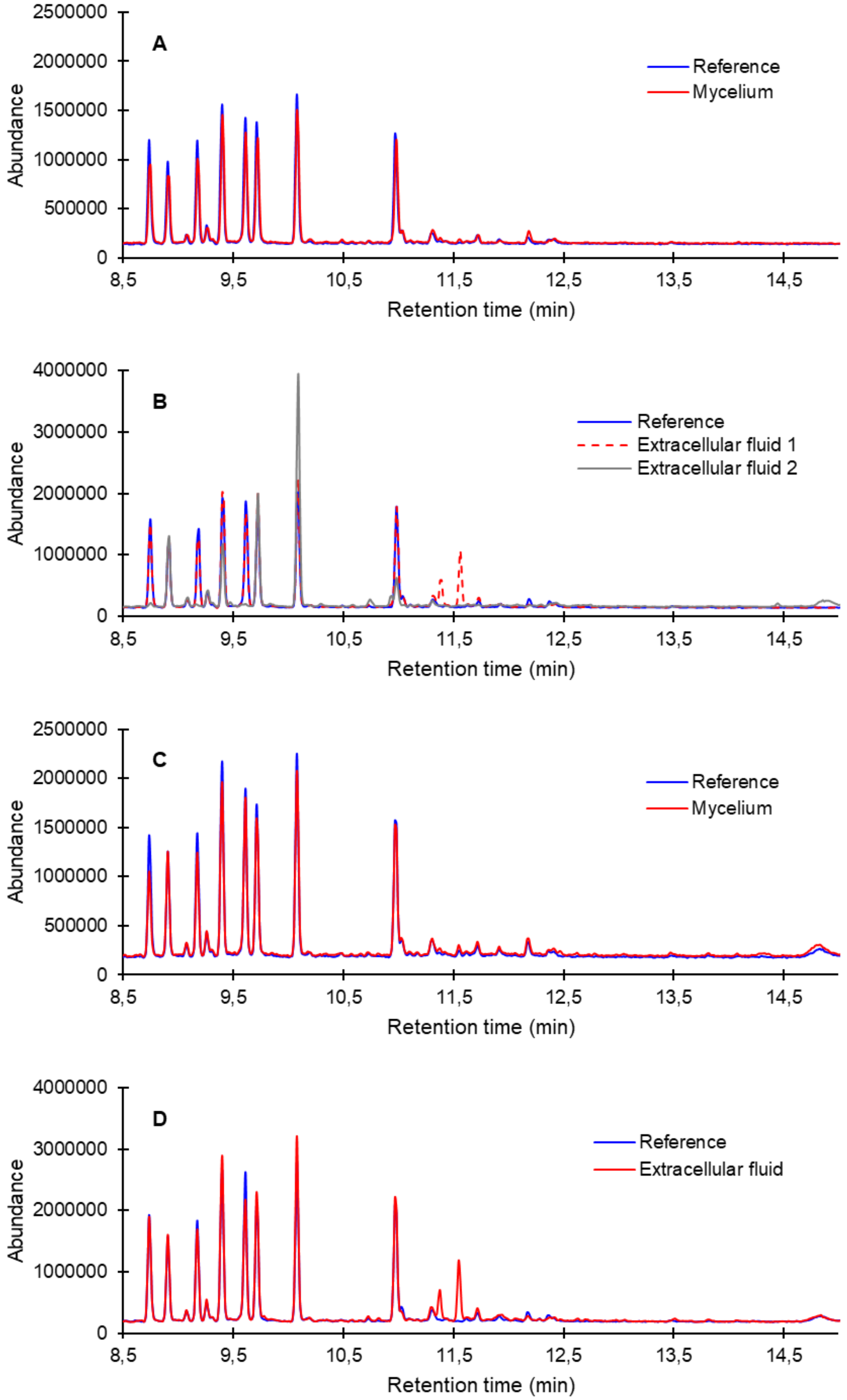

Supplementary Figure 7. Example GC-MS total ion chromatograms of the heartwood extract after incubation with fungal mycelium or extracellular fluid from sapwood-supplemented cultures vs. the

relevant reference. *C. puteana* mycelium (**A**), *C. puteana* extracellular fluid with and without degradation (**B**), *R. placenta* mycelium (**C**), *R. placenta* extracellular fluid (**D**)

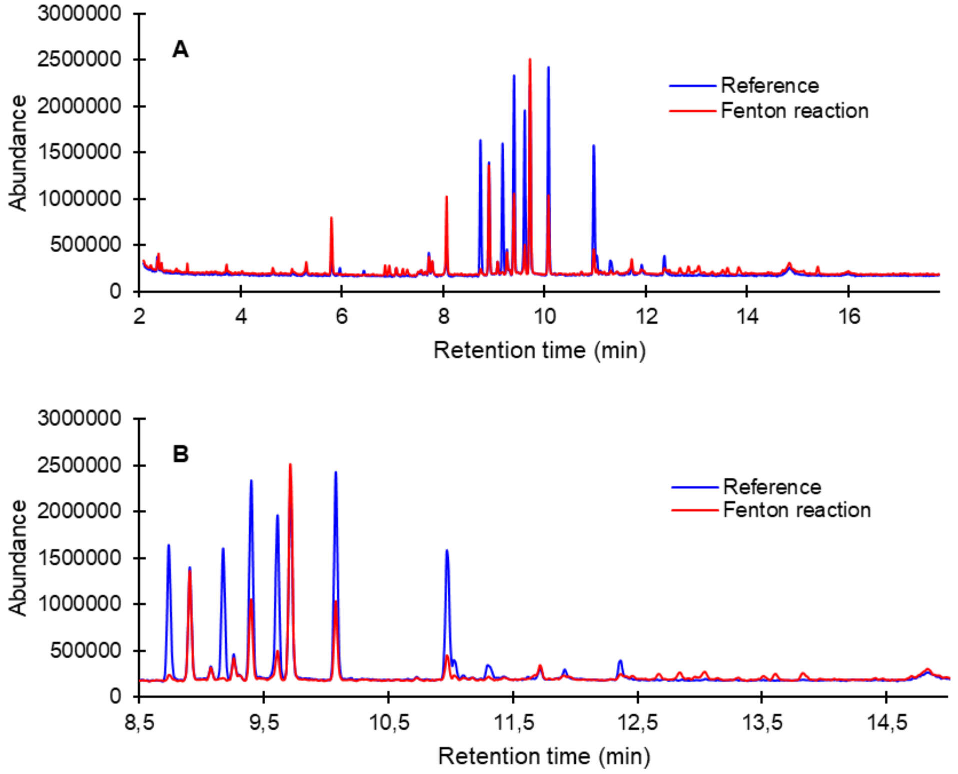

Supplementary Figure 8. Example GC-MS total ion chromatograms of the heartwood extract after incubation with Fenton reagent or with buffer (reference) from 2 to 17.8 min (**A**) and from 8.5 to 15 min (**B**)
